# Complete Genome Sequence of Microbacterium foliorum Bacteriophage Librie

**DOI:** 10.1128/mra.00918-22

**Published:** 2022-09-19

**Authors:** Sergei A. Markov, Nygil L. Arms, Kayla J. Boyce, Melody R. Cardona Pendleton, Angilena M. Couch, Leigh E. Duncan, Osamiabe I. Enodiana, Jaci N. Gibson, Kendall J. Greer, Claudine M. Habib, Ugonna G. Isaac, Tamia C. Johnson, Gabriella G. Lewis, Summer K. Long, Isela A. Ogas, Kehinde O. Olusoga, Patience O. Oni, Kim-Ngan H. Victory, Robin J. Zimmer

**Affiliations:** a Biology Department, Austin Peay State University, Clarksville, TN, USA; DOE Joint Genome Institute

## Abstract

Bacteriophage Librie was isolated from a soil sample from Clarksville, TN, using the bacterium Microbacterium foliorum. Librie has a 39,941 bp genome with 62 predicted protein-coding genes and 1 predicted gene for tRNA. Based on its gene content similarity to actinobacteriophages, Librie is grouped with phages in cluster EA5.

## ANNOUNCEMENT

**FIG 1:**
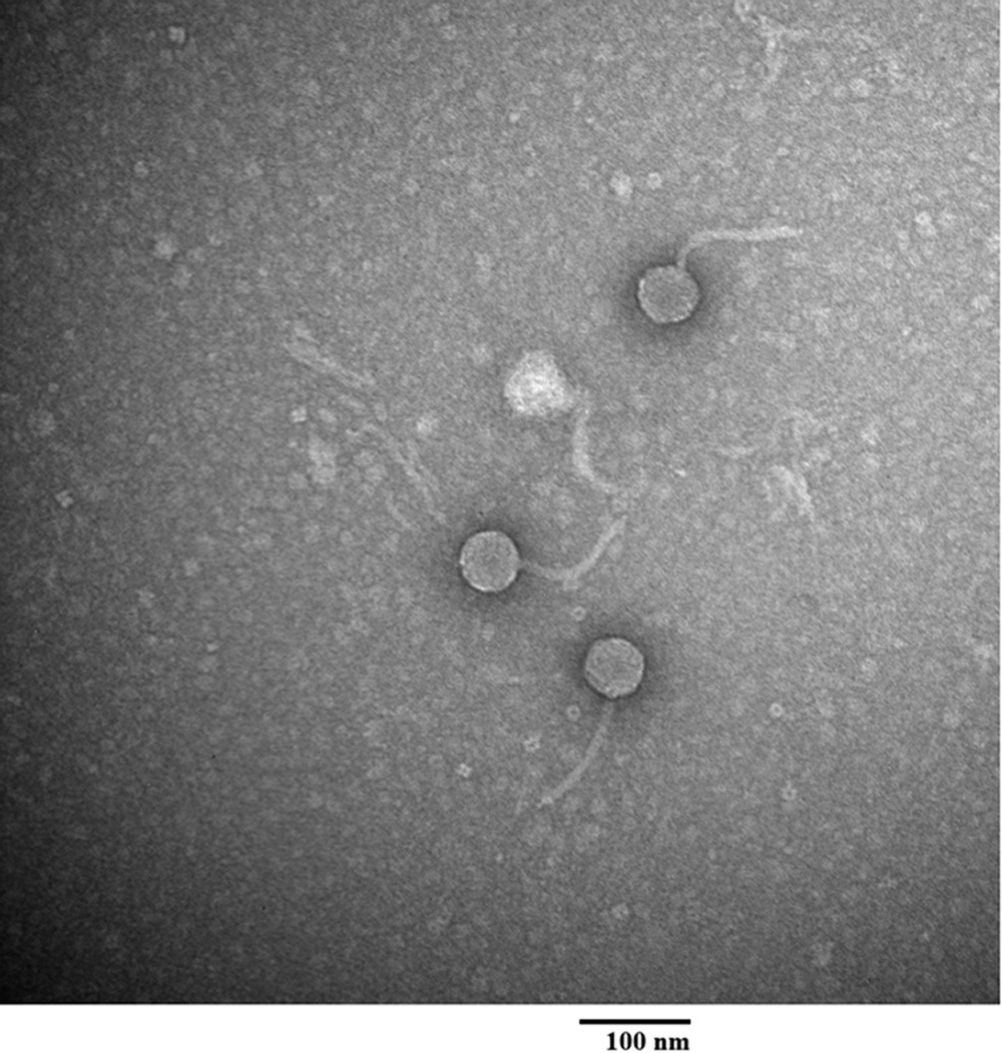
Transmission electron microscopy photo of Microbacterium foliorum bacteriophage Librie, a siphovirus with a flexible 132 to 134 nm long tail and an icosahedral 52 to 54 nm diameter capsid (*n* = 3). The bacteriophage sample was stained using 1% uranyl acetate on grids attached to Pelco Tabs (Ted Peller, Inc., Redding, CA). A Hitachi H-7650 Transmission Electron Microscope (Tokyo, Japan) was used for imaging with an accelerating voltage of 100 kV.

Expanding upon previously isolated bacteriophages from Tennessee, and as a means by which to further explore the genetic diversity and evolution of bacteriophages (1, 2), a new bacteriophage Librie was isolated on November 10, 2021, from a wet soil of Clarksville, Tennessee (36.531077° N, 87.357418° W), using a host bacterium Microbacterium foliorum NRRL B-24224 and standard procedures (https://seaphagesphagediscoveryguide.helpdocsonline.com/home). The soil sample was collected when the ambient temperature was 10°C, and the sample was suspended in peptone-yeast calcium (PYCa) liquid medium for 2 h. Subsequently, the suspension was passed through a 0.22-μm-pore filter, and the filtrate was inoculated with *M. foliorum* and incubated with shaking at 250 rpm for 2 days at 30°C. Following incubation, the culture was filtered, the filtrate was plated in PYCa top agar with *M. foliorum*, and the plates were incubated for 2 days at 30°C. The resulting phage, Librie, formed clear round plaques of 4 to 5 mm in diameter and was plaque purified through two rounds of plating. Negative stain transmission electron microscopy revealed Librie to possess a siphovirus morphology with a nonenveloped capsid and a flexible tail (Fig. 1).

DNA from Librie was isolated using the Promega Wizard DNA Clean-Up Kit and was prepared as a sequencing library using the NEB Ultra II Library Kit. The genome was then sequenced at the Pittsburgh Bacteriophage Institute using an Illumina MiSeq instrument (v3 reagents) to yield 698,002 150-base single-end reads and 20-fold coverage of the genome. Raw reads were assembled with Newbler v.2.9, and the resulting contig was checked for completeness using Consed v.29. The genomic termini were verified as described by Russell (3). The genome sequence of Librie was annotated using DNA Master v. 5.23.6 embedded with Glimmer v. 3.02 (4) and GeneMark v. 2.5p (5), PhagesDB BLAST (https://phagesdb.org/blastp/) (6), NCBI BLAST (7), HHPred v. 3.2 (8), Phamerator v. 393.0 (9), tRNAscanSE v. 2.0 (10), and PECAAN (http://pecaan.kbrinsgd.org/). We applied the default parameters for all software.

Bacteriophage Librie has a circularly permuted genome of 39,941 bp with a guanine-cytosine content of 65.3%. This genome contains 62 predicted protein-coding genes. We could assign a predicted function for 30 of the genes, and 1 gene was for tRNA (tRNA-Undet [nnn]). Based on its gene content similarity (GCS) of at least 35% to phages in the Actinobacteriophage database (phagesDB), using the GCS tool (https://phagesdb.org/genecontent/), the bacteriophage Librie is assigned to phage cluster EA5, where it is most closely related to the bacteriophage Hasitha (99% nucleotide identity), which was isolated in nearby Bowling Green, KY (6, 11). Currently, subcluster EA5 contains only eight bacteriophages.

The genome is organized similarly to cluster EA phages, with the left and right halves of the genomes encoding rightwards- and leftwards-transcribed genes, respectively (12). The left half contains genes for virion structure, assembly, and lysis, with a predicted programmed translational frameshift in the tail assembly chaperone genes (*16* and *17*). On the right half of the genome are genes involved in DNA metabolism, including DNA primase/helicase, RecA-like DNA recombinase, thymidylate synthase, and glycosyltransferase, as well as several genes with transmembrane domains. We did not identify any immunity repressors or integrase functions, and we predict Librie to be a lytic phage.

### Data availability.

The GenBank and SRA accession numbers for Librie are ON970570 (GenBank) and SRX14483211 (SRA), respectively.

## References

[1] Markov SA, Church JC, Lee L, Bell CM, Binkley SD, Bouma KM, Hutson KM, Markov GS, Mason EC, Rueff GB, Sennuga TO, Simpson MH, Zimmer RJ, Villalpando DG. 2021. Complete genome sequences of *Microbacterium* bacteriophages Danno, Otwor and Scumberland isolated in Clarksville. Microbiol Resour Announc 10:e00209-21. doi:10.1128/MRA.00209-21. 33795346 PMC8104054

[2] Hatfull GF. 2020. Actinobacteriophages: genomics, dynamics, and applications. Annu Rev Virol 7:37–61. doi:10.1146/annurev-virology-122019-070009. 32991269 PMC8010332

[3] Russell DA. 2018. Sequencing, assembling, and finishing complete bacteriophage genomes. Methods Mol Biol 1681:109–125. doi:10.1007/978-1-4939-7343-9_9. 29134591

[4] Delcher AL, Harmon D, Kasif S, White O, Salzberg SL. 1999. Improved microbial gene identification with GLIMMER. Nucleic Acids Res 27:4636–4641. doi:10.1093/nar/27.23.4636. 10556321 PMC148753

[5] Besemer J, Borodovsky M. 2005. GeneMark: web software for gene finding in prokaryotes, eukaryotes, and viruses. Nucleic Acids Res 33:451–454. 15980510 10.1093/nar/gki487 PMC1160247

[6] Russell DA, Hatfull GF. 2017. PhagesDB: the Actinobacteriophage Database. Bioinformatics 33:784–786. doi:10.1093/bioinformatics/btw711. 28365761 PMC5860397

[7] Altschul SF, Gish W, Miller W, Myers EW, Lipman DJ. 1990. Basic local alignment search tool. J Mol Biol 215:403–410. doi:10.1016/S0022-2836(05)80360-2. 2231712

[8] Söding J, Biegert A, Lupas AN. 2005. The HHpred interactive server for protein homology detection and structure prediction. Nucleic Acids Res 33:W244–W248. doi:10.1093/nar/gki408. 15980461 PMC1160169

[9] Cresawn SG, Bogel M, Day N, Jacobs-Sera D, Hendrix RW, Hatfull GF. 2011. Phamerator: a bioinformatics tool for comparative bacteriophage genomics. BMC Bioinformatics 12:395. doi:10.1186/1471-2105-12-395. 21991981 PMC3233612

[10] Lowe T, Chan P. 2016. tRNAscan-SE On-line: integrating search and context for analysis of transfer RNA genes. Nucleic Acids Res 44:W54–W57. doi:10.1093/nar/gkw413. 27174935 PMC4987944

[11] Pope WH, Mavrich TN, Garlena RA, Guerrero-Bustamante CA, Jacobs-Sera D, Montgomery MT, Russell DA, Warner MH, Hatfull GF, Science Education Alliance-Phage Hunters Advancing Genomics and Evolutionary Science (SEA-PHAGES). 2017. Bacteriophages of *Gordonia* spp. display a spectrum of diversity and genetic relationships. mBio 8:e01069-17. doi:10.1128/mBio.01069-17. 28811342 PMC5559632

[12] Jacobs-Sera D, Abad LA, Alvey RM, Anders KR, Aull HG, Bhalla SS, Blumer LS, Bollivar DW, Bonilla JA, Butela KA, Coomans RJ, Cresawn SG, D'Elia T, Diaz A, Divens AM, Edgington NP, Frederick GD, Gainey MD, Garlena RA, Grant KW, Gurney SMR, Hendrickson HL, Hughes LE, Kenna MA, Klyczek KK, Kotturi H, Mavrich TN, McKinney AL, Merkhofer EC, Moberg Parker J, Molloy SD, Monti DL, Pape-Zambito DA, Pollenz RS, Pope WH, Reyna NS, Rinehart CA, Russell DA, Shaffer CD, Sivanathan V, Stoner TH, Stukey J, Sunnen CN, Tolsma SS, Tsourkas PK, Wallen JR, Ware VC, Warner MH, Washington JM, Westover KM, et al. 2020. Genomic diversity of bacteriophages infecting *Microbacterium* spp. PLoS One 15:e0234636. doi:10.1371/journal.pone.0234636. 32555720 PMC7302621

